# New Conjugated Compound T5 Epidioxy-Sterol-ANB Inhibits the Growth of *Mycobacterium tuberculosis* Affecting the Cholesterol and Folate Pathways

**DOI:** 10.3389/fmicb.2020.537935

**Published:** 2020-09-10

**Authors:** Andres Baena, Emanuel Vasco, Manuel Pastrana, Juan F. Alzate, Luis F. Barrera, Alejandro Martínez

**Affiliations:** ^1^Grupo de Inmunología Celular e Inmunogenética, Facultad de Medicina, Universidad de Antioquia, Medellín, Colombia; ^2^Grupo de Productos Naturales Marinos, Facultad de Ciencias Farmacéuticas y Alimentarias, Universidad de Antioquia, Medellín, Colombia; ^3^Grupo de Parasitología, Facultad de Medicina, Universidad de Antioquia, Medellín, Colombia; ^4^Centro Nacional de Secuenciación Genómica, Facultad de Medicina, Universidad de Antioquia, Medellín, Colombia

**Keywords:** antimicobacterial, macrophages, cholesterol pathway, folic acid pathway, *Mycobacterium tuberculosis*

## Abstract

The upsurge and persistence of drug resistant strains of *Mycobacterium tuberculosis* (Mtb) is an important limitant to the battery of drugs available for the elimination of tuberculosis (TB). To avoid future scarcity of antibiotics against Mtb, it is important to discover new effective anti-mycobacterial agents. In this study, we present data from a series of experiments to determine *in vitro* and *in vivo* anti-mycobacterial activity of a library of epidioxy-sterol analogs. We test 15 compounds for their ability to reduce the viability of Mtb. We found that one compound called T5 epidioxy-sterol-ANB display significant potency against Mtb *in vitro* specifically inside macrophages but without effectivity in axenic cultures. A viability assay confirms that this T5 compound is less toxic for macrophages *in vitro* as compared to the current Mtb drug Rifampicin at higher concentrations. We use a transcriptomic analysis of Mtb inside macrophages after T5 epidioxy-sterol-ANB treatment, and we found a significant down-regulation of enzymes involved in the cholesterol and folic acid pathways. *In vivo*, significant differences were found in the lungs and spleen CFUs of Mtb infected mice treated with the T5 epidioxy-sterol-ANB as compared with the untreated control group, which provides additional evidence of the effectivity of the T5 compound. Altogether these results confirm the potential of this T5 epidioxy-sterol-ANB compound against Mtb.

## Introduction

Tuberculosis (TB), produced by *Mycobacterium tuberculosis* (Mtb), is the primary cause of death worldwide due to an infectious agent (McShane, 2019). In 2018, TB killed 1.5 million people, and 10 million were infected in the same year (World Health Organization [WHO], 2018). The worldwide prevalence of TB is sustained by the continuing HIV-AIDS pandemic, widespread antibiotic multi-drug-resistant (MDR) strains, and extensively drug-resistant (XDR) strains and poverty. Also, more than half of a million cases of rifampicin resistant TB were reported annually, with nearly 78% of those cases being MDR-TB. In addition, WHO statistics reported that about 1 in 3 deaths of TB are due to antimicrobial resistance (World Health Organization [WHO], 2018).

TB requires treatment with a combination of drugs; four anti-TB drugs taken for 6 months are required for the most drug-sensitive forms of TB (Lange et al., 2019). Treatment for MDR-TB and XDR-TB has been demonstrated to be lengthy and complicated; usually for XDR-TB patients the treatment consists of a combination of at least eight drugs, in some cases involving daily injections, for 18 months or even longer (Pontali et al., 2019). In 2019, the US Food and Drug Administration (FDA) approved the new BPaL regimen containing the drugs pretomanid, bedaquiline, and linezolid (Burki, 2019). In the Nix-TB trial in South Africa, this drug combination cured within just 6 months up to 90% of XDR tuberculosis with 5 pills a day (Nunn et al., 2019).

The development of new antibiotics by the pharmaceutical industry had mostly been stuck due to economic and regulatory hurdles (Spellberg, 2014; Anonymous., 2019). When new drugs against infectious diseases are used, the emergence of resistance is almost inevitable. Though bacterial evolution is unpredictable, the timeline for the development of resistance is uncertain. Different mechanisms have been associated with antibiotic resistance that includes mutations in genes targeted by antibiotics, the degradation or modification of antibiotics by the bacteria, the overexpression of efflux pumps to reduce the uptake of antibiotics, and alterations of the cell wall by osmoregulation in the phagosome (Larrouy-Maumus et al., 2016; Gygli et al., 2017; Laws et al., 2019). Intracellular persistence within macrophages is an essential feature of Mtb pathogenesis (Baena and Porcelli, 2009; Queval et al., 2017). When Mtb is inside macrophages it normally replicates in phagosomes, which are believed to be a restricted and stressful environment (Ehrt and Schnappinger, 2009; Bussi and Gutierrez, 2019). To be able to replicate in this closed environment, Mtb utilizes particular metabolic pathways to obtain host-derived nutrients (Lopez-Agudelo et al., 2017; Baena et al., 2019; Mashabela et al., 2019). A variety of transcriptional profiling studies in macrophages have indicated that lipid metabolism and cholesterol are important for Mtb survival (Aguilar-Ayala et al., 2017; Zimmermann et al., 2017; Baena et al., 2019; Lee et al., 2019). Additionally, genes involved in cholesterol utilization, gluconeogenesis, or the methyl citrate cycle (MCC) are required for full Mtb virulence during infection; mutants in some genes along these metabolic pathways fail to establish infection in macrophages (Joshi et al., 2006; Pandey and Sassetti, 2008; Miner et al., 2009; Russell, 2011; Griffin et al., 2012; Lopez-Agudelo et al., 2017; Fieweger et al., 2019). Based on this information, the central carbon metabolic pathways of Mtb are thought to be potential targets for TB drug discovery. An important study showed a structurally diverse set of compounds that target the Mtb cholesterol pathway that causes growth restriction of the bacteria inside macrophages but not in axenic cultures in the absence of cholesterol (VanderVen et al., 2015).

In summary, it is vital to unveil new drugs to treat TB that inhibit new biological targets and pathways. Identifying small molecules that are capable of inhibiting specific enzymatic targets in Mtb using target-based screens is still challenging. In this report we used a chemical library screen to identify new compounds that inhibit Mtb replication during infection inside macrophages, which allow us to identify the T5 epidioxy-sterol-ANB compound with a good anti-mycobacterial activity. The T5 compound is the result of the conjugation of the 4-nitrobenzoic acid (ANB) and the 5α,8α-epidioxy-3β-cholesterol. Although there are a few reports in the literature showing some activity of these molecules, none of them have been used *in vivo* in animal models for tuberculosis (Cateni et al., 2007; Giampaglia et al., 2007). Here we show *in vivo* activity and a metabolic effect of this T5 compound that suggest that it may inhibit simultaneously the cholesterol degradation and folic acid synthesis pathways in Mtb.

## Materials and Methods

### Bacteria and Plasmid

H37Rv was grown at 120 rpm in a shaker at 37°C in a square bottle containing 10 ml of 7H9 (Difco, Sparks, MD. United States) supplemented with 10% of oleic acid-albumin-dextrose-catalase (OADC) (Becton Dickinson Microbiology Systems, NJ, United States), 0.5% Glycerol (Sigma, Saint Louis, MO, United States), and 0.05% Tyloxapol (Sigma, Saint Louis, MO, United States), to an optical density of 0.5 at OD_600 nm_. H37Rv-pMV261.kan-GFP was grown in the presence of 50 μg/ml of kanamycin. The pMV261.kan-GFP is a multicopy plasmid under the control of the hsp60 that expresses GFP.

### Compound Library Synthesis

Syntheses of the 5 epidioxy steryl esters were done in two steps. The first step is the synthesis of the compound 1, 5α,8α-epidioxy-3β-cholesterol, by the photochemical reaction of 7-dehydrocholesterol with oxygen mediated by eosine like *photosensitizer* (Feng et al., 2007). In this reaction the endoperoxide group was formed in positions C-5 and C-8 in a [4 + 2] cycloaddition reaction. The aromatic esters were done by the Steglich methodology with compound 1 using DCC and DMAP in CHCl_3_ with different aromatic acids (Farshori et al., 2010) (Supplementary Figure S1).

### NMR of Compound T5

The NMR experiments were obtained in CDCl3 using a Bruker spectrometer AVANCE III NMR operating at 600 MHz for 1H and 150 MHz for 13C.

^1^H NMR (600 MHz, Chloroform-d) δ 9.22 (d, J = 2.1 Hz, 1H), 9.14 (d, J = 2.2 Hz, 2H), 6.56 (d, J = 8.5 Hz, 1H), 6.29 (d, J = 8.5 Hz, 1H), 5.36 (q, J = 11.2, 5.1 Hz, 1H), 2.31 (ddd, J = 13.6, 5.6, 1.7 Hz, 1H), 2.26 (dd, J = 13.6, 11.6 Hz, 1H), 2.16 – 2.07 (m, 2H), 2.05 – 1.98 (m, 1H), 1.98 – 1.89 (m, 1H), 1.85 – 1.74 (m, 2H), 1.65 (m, J = 11.6, 9.0, 8.1, 2.5 Hz, 1H), 1.61 – 1.49 (m, 4H), 1.49 – 1.39 (m, 1H), 1.41 – 1.36 (m, 1H), 1.39 – 1.30 (m, 2H), 1.30 – 1.17 (m, 3H), 1.14 (s, 2H), 1.19 – 1.07 (m, 2H), 1.06 – 0.98 (m, 1H), 0.98 (s, 3H), 0.91 (d, J = 6.5 Hz, 3H), 0.87 (dd, J = 6.7, 2.9 Hz, 7H), 0.85 (s, 3H), 0.82 (s, 3H).

^13^C NMR (151 MHz, Chloroform-d) δ 148.61, 134.70, 131.24, 129.45, 122.29, 81.75, 79.62, 72.74, 56.39, 51.47, 50.97, 44.77, 39.42, 39.35, 36.99, 35.93, 35.22, 34.27, 33.13, 28.24, 27.99, 26.30, 23.78, 23.42, 22.81, 22.55, 20.60, 18.57, 18.12, 12.65.

### Cell Viability Assay

The AMJ2-C8 macrophages were infected at an MOI of 10:1. Cell viability was determined by fluorescence microscopy. After 48 hrs of treatment with the compound library, the treated cells were stained with Acridine Orange (AO) and Propidium Iodide (PI) in PBS (4 mg/ml). AO is excited at wavelengths near 502 nanometers (nm) when intercalated with dsDNA, emitting a green fluorescence with wavelengths of approximately 525 nm. AO stains acidic compartments such as lysosomes, where it becomes sequestered and protonated in live cells. Within this low lysosomal pH, the vesicles emit red fluorescence when loaded with the dye. When cells have an affected plasma membrane integrity, the PI is permeable and emits red fluorescence in the nucleus once it is intercalated with the DNA, which is excited at wavelengths of approximately 535 nm and fluoresces in the spectrum of 617 nm. Thus, cell viability could be assessed by the differential uptake of both dyes. The method for the cell viability quantification is showed in Supplementary Figure S4.

### MTT Assay

The MTT (3-[4,5-dimethylthiazol-2-yl]-2,5 diphenyl tetrazolium bromide) assay is founded on the conversion of MTT into formazan crystals by viable bacteria, which determines succinate dehydrogenase activity. The MTT assay was carried out as described before (Mshana et al., 1998). Briefly, in a 96 well plate we put 5 × 10^7^ bacteria (H37Rv) with 40 μL of culture medium into each well, and then each compound was added at different concentrations diluted with DMSO. We performed three independent experiments for each compound concentration. The plates were incubated at 37°C for 48 h. The compound MTT (Sigma, St. Louis, MO, United States) was dissolved in PBS (pH 7.2) to get a final concentration of 5 mg/ml. Following, 10 μL of the MTT solution was added to each well in the plate and incubated for 4 h at 37°C. Finally, 50 μL of a lysing buffer (20% sodium dodecyl sulfate in 50% *N1N*-dimethylformamide [pH 4.7]) was added to each well, and the plates were incubated overnight. The absorbance was measured with a spectrophotometer at a wavelength of 570 nm. The H37Rv bacteria was grown as described above in the bacteria and plasmid section. CFU counts of H37Rv axenic culture after 48 h of treatment with the T5 and Rifampicin compounds were determined in 7H10 agar plates.

### Macrophage Cell Lines and Mtb Infection

The AMJ2-C8 murine alveolar macrophage cell line was obtained as a gift from Steven A. Porcelli at the Albert Einstein College of Medicine. The cells were grown in DMEM supplemented with 10% FBS, 1X Pen/strep, 0.05 mM of 2-mercaptoethanol, and 1X NEAA.

The THP-1 cell line was obtained from ATCC (TIB-202) and maintained in sterile RPMI-1640 medium supplemented with 10% FBS, 1X Pen/strep, 0.05 mM of 2-mercaptoethanol, and 1X NEAA. For differentiation 2 × 10^6^ THP-1 cells were seeded per well in a 6-well plate and differentiated to macrophages for 48 h, in the presence of 100 ng/ml PMA (162 nM). After a resting period of 24 h, macrophages were then infected with Mtb H37Rv for 4 h at a multiplicity of infection (MOI) of 10:1, and incubated for 4 and 20 h (RNA-seq experiments) and also for 48 h (CFUs experiments in the presence of the T5 and rifampicin compounds), at 37°C with 5% CO_2_. To measure the survival of the cell-associated Mtb-H37Rv, THP-1 cells were lysed in 1.5 mL of distilled water with 1% SDS to collect the intracellular bacteria. The lysates were serially diluted in 7H9 broth, plated on 7H10 agar plates, and incubated for 3 weeks at 37°C. Colony counting was then performed in triplicate.

### *In vivo* Experiment With the T5 Compound

Female wild type Balb/C mice (6–8 weeks of age) were used in the experiment and maintained under a sterile BSL3 facility. Four mice per group were infected with 2 × 10^5^ bacteria intra peritoneal (i.p) in 200 μL of PBS with 0.05% Tween 80. The amount of bacteria during the challenge was verified by plating the inoculum on 7H10 plates. After 4 days of infection, the mice were treated with the T5 compound or the vehicle. The mice received 550 μg/200 μL of T5 compound daily by i.p injection (25 mg/Kg). On day 32, the mice were sacrificed, and the lungs and spleens were smashed and plated in 7H10 media plates. After 24 days, the CFUs were estimated. All experiments were performed in accordance with the Ethics Committee for Animal Experiments (CEEA) of the University of Antioquia.

### RNA Extraction

The Mtb infected THP-1 cells were lysed at 4°C by adding 1 ml of RLT buffer (RNeasy Plus mini kit, QIAGEN, Hilden, Germany). We maintained the lysates frozen in liquid nitrogen for 15 s and then the lysates were homogenized for 20 s with a tissue tearor homogenizer (Biospec, Bartlesville, OK, United States; model 985–370 at 5,000 rpm). The homogenization procedure was repeated two times with an intermediate incubation of 1 min on ice. Next, the lysates were transferred to an Eppendorf 1.5-ml tube where they were disrupted using bead beating (Bead beater Instrument; applying six cycles of 30 s at maximum speed with cooling on ice between the different cycles) using high impact zirconium-silica beads (BenchmarkScientific, Bartlesville, OK, United States). Following this, the samples were centrifuged at 10,000 rpm for 10 min at 4°C. Finally, we used the aqueous phase to extract the total RNA with the Qiagen RNeasy plus mini kit, which includes the DNA retention column according to the manufacturer’s instructions. We performed three independent experiments for each condition, and the extracted RNA was pooled for its related replicates. The RNA Integrity Number (RIN) was determined by a Bioanalyzer 2000 instrument; values for all the samples were between 8.1 and 8.6.

### RNA-seq

RNA-seq libraries were sequenced using an Illumina platform (Macrogen, South Korea). The libraries for the RNA-seq were generated using the TruSeq^®^RNA-Sample-Preparation Kitv2 following the manufacturer instructions (Illumina, Inc., San Diego, CA, United States). Briefly, the sequencing library is prepared by random fragmentation of the RNA sample, followed by 5′ and 3′ adapter ligation. The libraries started with double-stranded cDNA synthesized from RNA with insert sizes from 300–500 bp that are used for paired-end sequencing. Blunt-end DNA fragments are produced using a combination of fill-in reactions and exonuclease activity. An ‘A’- base is then added to the blunt ends of each strand, preparing them for ligation to the sequencing adapters. Each adapter contains a ‘T’-base overhang on 3′-end providing a complementary overhang for ligating the adapter to the A-tailed fragmented DNA. These newly redesigned adapters contain the full complement of sequencing primer hybridization sites for single, paired-end, and multiplexed reads. rRNA from macrophages and bacteria was depleted (Illumina Ribo-Zero Gold rRNA Removal Kit -Epidemiology), and sequencing was performed (TrueSeq, stranded, paired-end reads of 100 bp). The “clean” read dataset (clean reads refer to reads that passes the process of adapter removal, low quality bases removal at both ends [<Q30] and that after this two process the reads retain a minimum length of 70 bases) was used in order to map to the reference genome of the H37Rv strain of Mtb (AL123456_update130713), using the software BOWTIE2 with the default settings. We converted the SAM file to BAM, and then we sorted and indexed the reads using SAMTOOLS. Reads counts assigned to each gene present in the reference Mtb strain were done by means of the HTSEQ script with the stranded option. Next, we used the R package EDGER for the differential expression analysis, following the directions of the software authors for the different RNA-seq experiments. We stablished the coefficient of biological variation to 0.3, due to the lack of library replicates. The results of the EDGER analysis were printed as graphs and tables. The RNA-seq transcriptomic data was deposited in https://www.ncbi.nlm.nih.gov/bioproject: DMSO 4 h, BaenaRNA1_1.fastq.gz (SRR11195990); DMSO 20 hrs, BaenaRNA3_1.fastq.gz (SRR11195989); T5 4 h, BaenaRNA2_1.fastq.gz (SRR11195988); and T5 20 h, BaenaRNA4_1.fastq.gz (SRR11195987).

## Results

### The T5 Compound Has Anti-mycobacterial Activity

**FIGURE 1:**
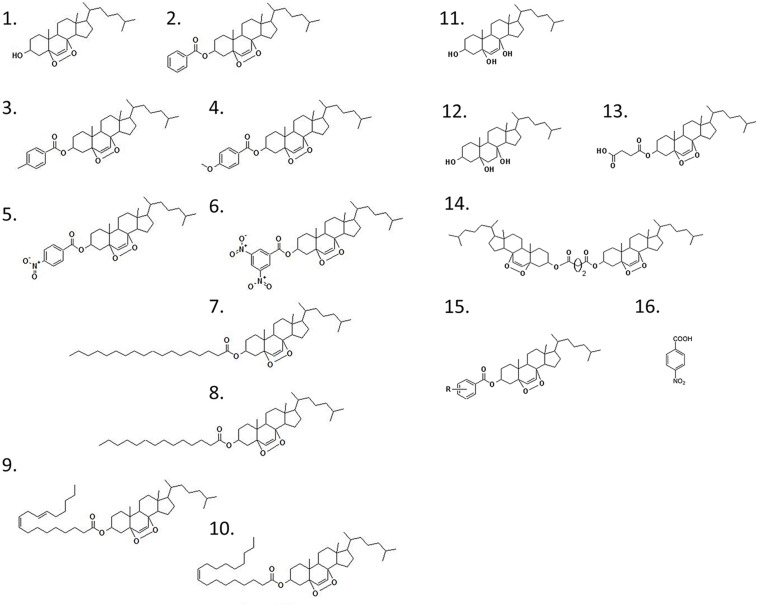
Chemical structure of the epidioxy-sterol analogs. This compound library started with the compound 1 (T1) that corresponded to a 5α,8α-epidioxy- 3β-cholesterol. The other compounds are called after the addition of a second (R) group from which we have generated the compounds 1–15 that in this paper we called T1–T15. The compound 16 is the 4-nitrobenzoic acid (ANB).

**FIGURE 2:**
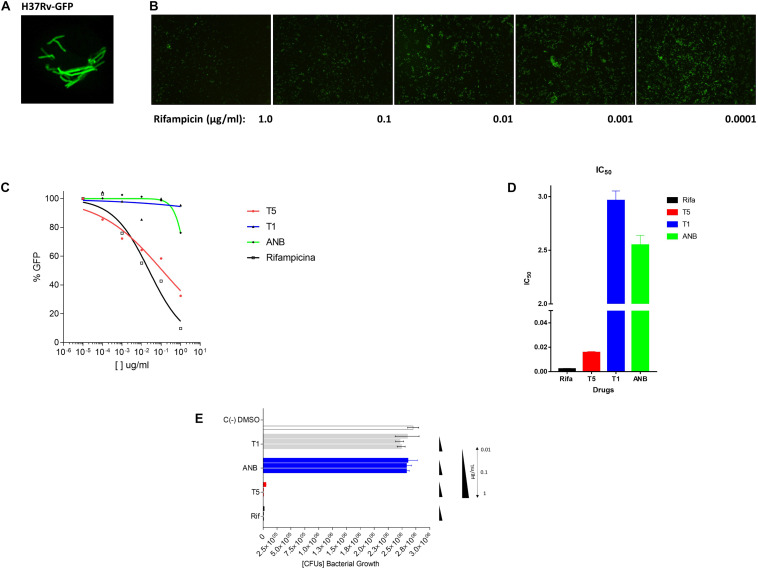
Inhibitory assay for the selection of compound analogs with activity against Mtb. **(A)** The Mtb bacteria H37Rv expressing the plasmid pMV261-GFP. **(B)** Inhibitory effect of Rifampicin (48 h) in the expression of GFP after the Mtb infection (4 h) of the THP-1 macrophage cell line. The panel shows 5 concentrations of the rifampicin compound. **(C)** Inhibitory GFP curves for the compound T5 relative to the compound T1, ANB, and the control compound rifampicin after 48 h of treatment post-infection. **(D)** Inhibitory concentration 50 (IC_50_) calculated based on the inhibitory curves. **(E)** The colony forming units (CFUs) that resulted after the lysis of the infected THP-1 cell line. The molecular weight (g/mol) of the compounds is as follow: T1 (416.6), T5 (565.7), ANB (167.11), and rifampicin (822.94). The equivalent molar concentration to the μg/mL used in the assays (1, 0.1, 0.01, 0.001, and 0.0001) for the different compound are: T1 (2.4, 0.24 μM, 24, 2.4, and 0.24 nM), T5 (1.7, 0.17 μM, 17, 1.7, and 0.17 nM), ANB (6, 0.6 μM, 60, 6, and 0.6 nM), and for rifampicin (1.2, 0.12 μM, 12, 1.2, and 0.12 nM).

**FIGURE 3:**
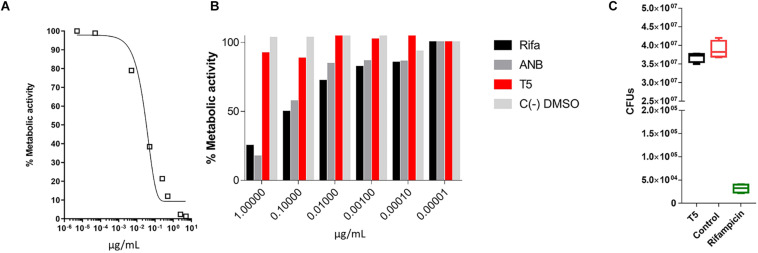
MTT assay to evaluate the inhibitory capacity of the T5 compound against an axenic culture of Mtb. **(A)** Evaluation of the% metabolic activity of the control compound rifampicin by the MTT assay *in vitro* for 48 h, against an axenic culture of Mtb. **(B)** Determination of the % metabolic activity based on the MTT assay of the T5 compound relative to the control compounds ANB and rifampicin at different concentrations. **(C)** CFU counts of H37Rv axenic culture after 48 h of treatment with the T5 and Rifampicin compounds. The optical density values (OD) obtained in the MTT assay were converted to percentage of metabolic activity relative to the DMSO control treatment which represent 100% of metabolic activity.

Previous reports have shown that epidioxy-sterols-like compounds have activity against Mtb (Saludes et al., 2002; Cateni et al., 2007; Duarte et al., 2007; Zhou et al., 2015). Thus, we decided to evaluate the activity of a unique library of 15 epidioxy-sterol analog conjugated compounds against this bacterium (Figure 1). For this purpose, we implemented an assay using H37Rv containing a plasmid that expresses GFP under a constitutive promoter. In this assay, Mtb replicates in the THP-1 macrophages and constitutively produces GFP. In the presence of an anti-mycobacterial compound GFP fluorescent signal is quenched in a concentration-dependent manner, as we observed with the positive control drug Rifampicin (Figures 2A,B). A similar strategy was previously used in the discovery of novel inhibitors of cholesterol degradation pathway in Mtb (VanderVen et al., 2015). The only compound that showed good activity in the infected THP-1 macrophages was the T5 compound, evidenced in the low IC_50_ of 0.116 μg/mL (0.2 μM) although 6.7 times higher than rifampicin that showed an IC_50_ of 0.024 μg/mL (0.03 μM) (Figures 2C,D). As we mentioned above, this T5 compound is the result of conjugation of the compound T1 and ANB. In contrast to T5, the compound T1 and ANB showed very low activity against Mtb (Figures 2C,D). Similar results were obtained by using the murine alveolar macrophage cell line AMJ2-C8 (data not showed). To confirm this result, we evaluate the Mtb viability by lysing the macrophages and plating the recovered bacteria and counted CFUs. The data shows undetectable CFUs at as low as 0.1 μg/ml, which is comparable to the treatment of Rifampicin (Figure 2E and Supplementary Figure S2). Thus, both the sterol ring and the ANB together in the T5 compound are required for effective anti-mycobacterial activity. We next analyzed the effectivity of the library compounds against an axenic Mtb culture. While the ANB compound has an anti-mycobacterial activity, the T5 compound did not show any significant activity under this experimental condition (Figures 3A,B). We confirmed this result with the CFU measurement in which rifampicin is able to reduce the bacterial numbers significantly but the result with the T5 compound is similar to the negative control (Figure 3C). The ANB also known as 4-nitrobenzoic acid was previously shown to have anti-mycobacterial activity against Mtb in axenic cultures in the diagnostic setting, but there is no data showing if the compound is effective inside macrophages (Giampaglia et al., 2007). Thus, we have a T5 compound with effective anti-mycobacterial activity inside macrophages but with no activity against extracellular bacteria. This could suggest that a particular processing condition inside the macrophage (e.g., activating enzymes) may be required to transform the T5 compound in order to work against Mtb.

### The T5 Compound Has Low Cell Toxicity at Higher Concentrations

**FIGURE 4:**
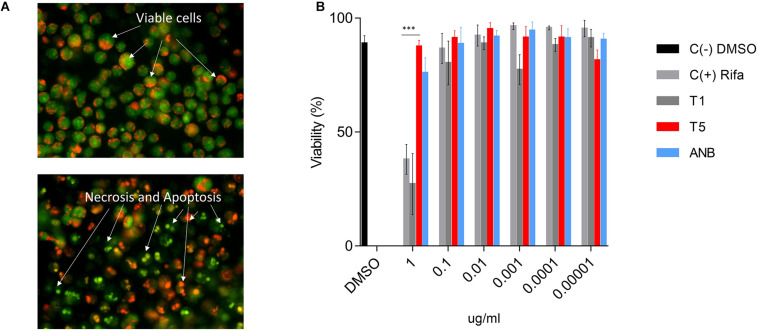
Cytotoxicity assay for T5 compound in the AMJ2-C8 cell line. **(A)** A representation of healthy and viable cells (no treatment) and cells treated with a high concentration of rifampicin (1 μg/mL). The cells were stained with acridine orange (AO) and propidium iodide (PI). **(B)** Viability (%) of the AM cell line after 48 h treatment with the T5 compound relative to the control compounds T1, ANB, and Rifampicin. ^∗∗∗^*p*-value < 0.0001. We are showing data of a representative experiment from four independent ones.

Cytotoxicity of the T5 compound was tested using the AMJ2-C8 macrophage cell line by staining the cells with AO and PI (Figure 4A). We used a range of concentrations (0.0001, 0.001, 0.01, 0.1, and 1 μg/ml) of the T5 as well as for T1 and ANB compounds, Rifampicin and DMSO, as the positive negative controls respectively. We found that while the T1 and Rifampicin are highly toxic at the higher concentration of 1 μg/mL, the T5 compound showed low cytotoxicity at the same concentration (Figure 4B). This is an interesting result because it is expected that any new candidate compound has low toxicity, which could be associated with fewer side effects for future experiments such as the experimentation in animal models.

### The Transcriptome of Infected THP-1 Cells Treated With the T5 Compound

**FIGURE 5:**
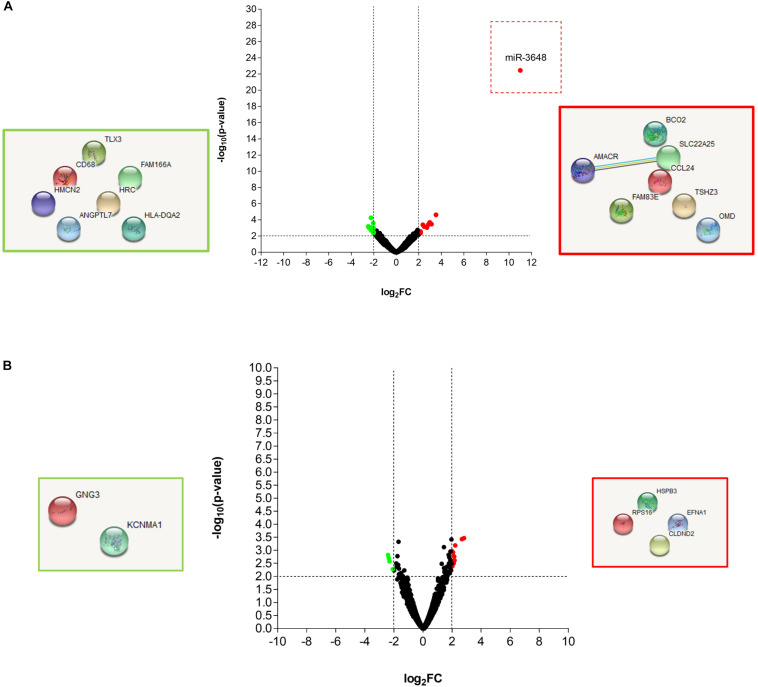
RNA-seq analysis of the THP-1 macrophages Mtb infected, and treated with the T5 compound. **(A)** Volcano plot and STRING analysis of the up-regulated and down-regulated genes at 4 h with a log2FC > 2.0 or log2FC < 2.0 and a *p*-value < 0.05. **(B)** Volcano plot and STRING analysis of the up-regulated and down-regulated genes at 20 h with a log2FC > 2.0 or log2FC < 2.0 and a *p*-value < 0.05.

In order to precisely define the genes in Mtb and in the THP-1 macrophages, which are affected by the T5 compound, we performed a dual transcriptome by RNA-seq. The macrophages were infected for 4 and 20 h and then the total RNA was extracted and sequenced (methods section, Supplementary Figure S3). Using log_2_FC > 2.0, log_2_FC < -2.0, and a *p*-value < 0.05 as selection criteria for differentially expressed genes, we found that macrophages expressed very few genes up-regulated and down-regulated in response to the T5 compound either at 4 or 20 h post-treatment (Figures 5A,B). The most noticeable up-regulated gene at 4 h was miR-3648 with a log_2_FC > 10 (Figure 5A). This gene was shown to be involved in the negative regulation of the adenomatous polyposis coli 2 (*APC2*) that is expressed in different cell lines and is a tumor suppressor gene (Rashid et al., 2017). Thus, the reduced levels of *APC2* may result in an increased expression of the Wnt/β-catenin signaling pathway genes, and promotes cell proliferation. Another interesting up-regulated gene is the BCO2 that encodes for a β-carotene-9′, 10′-oxygenase 2 (Figure 5A). The increased expression of BCO2 may confer and increase survival since its knockdown causes an increment in apoptosis due to its protective role to oxidative stress (Ref: Lei woo, Exp Biol Med [Maywood], 2016). Also, we have the up-regulation of the EFNA1 gene that codes for the Ephrin A1 protein, which is a member of the A-type ephrin family and has been implicated as a negative inductor of apoptosis (Ref: Spencer Alford, Cancer Cell Int. 2010). The up-regulation of the genes miR-3648, BCO2, and EFNA1 by the T5 compound could be associated with its lower cytotoxicity.

### Transcriptome of Mtb Treated With the T5 Compound Affects the Cholesterol and Folic Acid Pathways

**FIGURE 6:**
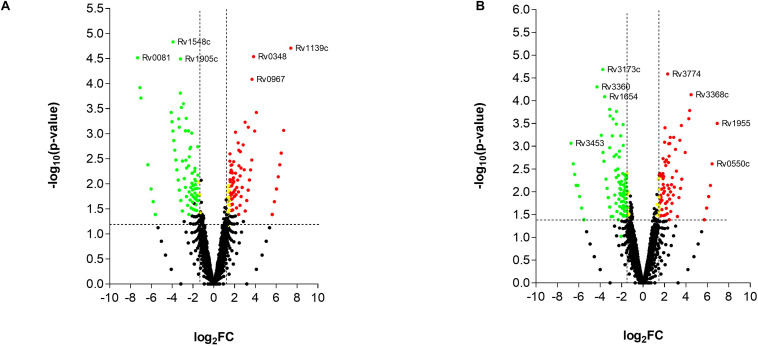
RNA-seq analysis of Mtb after the treatment with the T5 compound. **(A)** Volcano plot analysis of the up-regulated and down-regulated genes at 4 h with a log2FC > 2.0 or log2FC < 2.0 and a *p*-value < 0.05. **(B)** Volcano plot analysis of the up-regulated and down-regulated genes at 20 h with a log2FC > 2.0 or log2FC < 2.0 and a *p*-value < 0.05.

**FIGURE 7:**
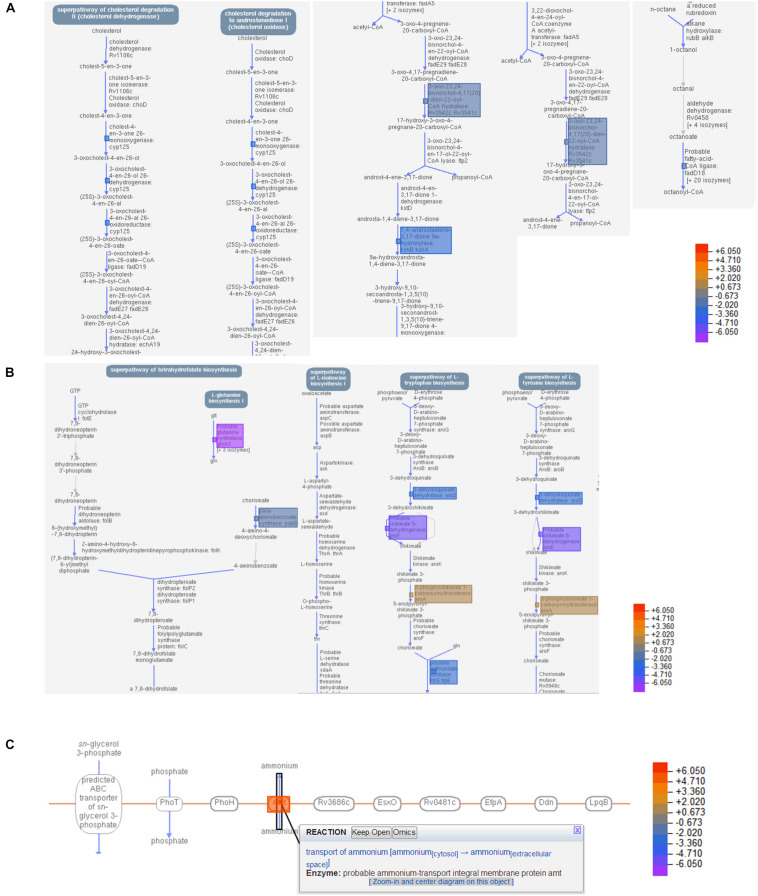
T5 compound affect the cholesterol and folic acid pathways in Mtb. **(A)** Shows the main enzymes and reactions of the cholesterol degradation pathway in Mtb that were significantly affected by the treatment of the T5 compound using the BioCyc database Collection (https://biocyc.org/organism-summary? object=MTBH37RV, version 23.1). **(B)** Shows the main enzymes and reactions of the folic acid synthesis pathway that were significantly affected by the treatment of the T5 compound using the BioCyc database. **(C)** Shows the up-regulation of the ammonium-transport integral protein (Amt) in response to the treatment with the T5 compound using the BioCyc database. The data in **(A,B)** are from the 4 h and in **(C)** from 20 h transcriptome.

While the transcriptome of the macrophage remained mostly unaffected by the treatment of the T5 compound, the Mtb transcriptome showed a significant number of up-regulated and down-regulated genes (Figures 6A,B). To find out which were the most affected pathways in Mtb under the treatment of the T5 compound, we used the Mtb pathway/genome from the BioCyc database collection^1^ (version 23.1). Using this approach, we found a significant down-regulation of enzymes involved in the cholesterol and folic acid pathways like the para-aminobenzoate synthase (pabB) (Figures 7A,B). In addition, we found a significant up-regulation of the ammonium-transport integral protein (*Amt*), which is important for detoxification of the bacteria from the accumulation of ammonia (Figure 7C).

### The Compound T5 Reduces the Mtb CFUs in the Mouse Model

**FIGURE 8:**
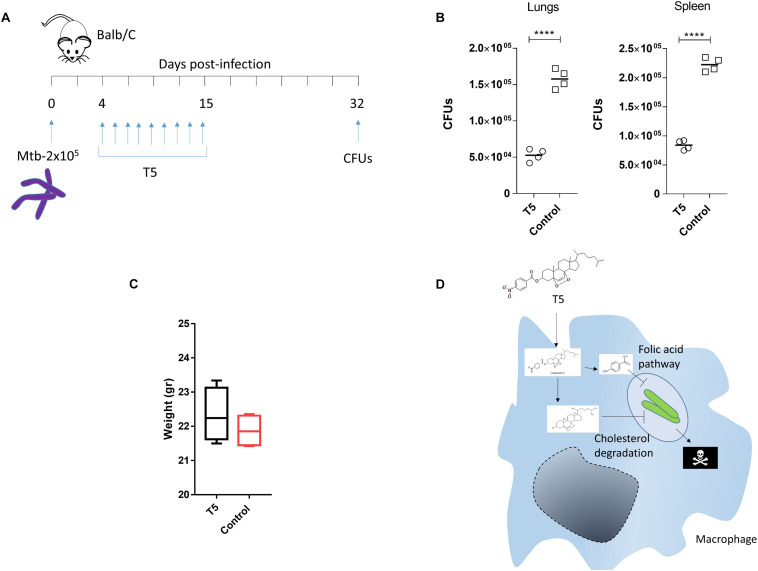
The T5 compound has anti-mycobacteral activity *in vivo*. **(A)** The diagram shows the treatment scheme for the T5 compound and the CFU determination time points. Four mice per group were infected with 2 × 10^5^ bacteria intra peritoneal (i.p) in 200 μL of PBS with 0.05% Tween 80. **(B)** Quantification of the CFUs in spleen and lungs of the T5 treated Balb/C mice after 32 days. **(C)** Body weight of Balb/C mice at the end of drug treatment 15 days post-infection. **(D)** Diagram showing a model of the possible mechanism of action of the T5 compound against Mtb. Unpaired *t*-test ****p-value < 0.0001. Representative of two independent experiments.

To validate the anti-mycobacterial activity of the T5 compound, we did a pilot *in vivo* experiment using Balb/C mice. In this experiment we observed that the T5 compound cause a significant 2–3 fold reduction of the Mtb CFUs in the lungs as well as in the spleen of the treated mice, as compared to the control vehicle treated group (Figures 8A,B). We did not observe significant differences in weight of the animals at the end of the treatment and 15 days post-infection between the T5 treated mice and the control group (Figure 8C). In summary, these findings suggest that this T5 compound is not only active *in vitro* but also *in vivo*.

## Discussion

One difficulty in TB drug discovery arises from a limited knowledge of the physiological environment and growth conditions that Mtb experienced during infection. A rising in drug-resistant TB is a major impediment to successfully deal with this disease. The WHO recognizes antimicrobial resistance as one of the most demanding global threats to cure TB. Drug resistance is probably a consequence of overuse of antibiotics in the treatment of humans or lack of adherence to the treatment. When a new antibiotic is introduced, it can have great results for a period of time, but then the bacteria gradually adapt and the antibiotic becomes less effective. This is why we need to maintain a constant search for new effective antibiotics that target different pathways in an organism like Mtb.

Our results define the activity of a new compound called T5, which has anti-mycobacterial activity against Mtb, specifically when the bacteria locate inside the macrophages, but surprisingly not in the extracellular environment. We hypothesize that this T5 compound may have a combinatorial effect in which the conjugation may help their entry into cells, likely due to the requirement of Mtb to utilize cholesterol. Once inside of the bacteria, an enzymatic reaction, probably mediated by a macrophage specific esterase, could release the ANB part from the T1 compound, and then the ANB may interfere with the enzymes of the folic acid pathway. In addition, the released T1 part of the T5 compound may simultaneously affect the enzymes of the cholesterol degradation pathways (Figure 8D).

Even though anti-folate drugs have been very successful as anticancer and antimicrobial agents, they are poorly effective in the TB therapy (Minato et al., 2015a; Hajian et al., 2019). The ineffectiveness of these type of drugs is probably due to the lack of permeability to them in Mtb (Nixon et al., 2014). Moreover, anti-folates have a past history of use in TB therapy especially through the use of the drug para-aminosalicylic-acid (PAS) that worked as a bacteriostatic anti-tubercular agent. Finding a compound that is both permeable and effective to inhibit this folate pathway would be highly desirable. On the other hand, the cholesterol catabolic pathway is an important therapeutic target in Mtb in which many of its genes are involved in pathogenesis. This has been demonstrated by the mutation or deletion of these genes and the attenuation of the bacteria. The cholesterol catabolic pathway in Mtb is accomplished in two major lanes, first by the degradation of the aliphatic side chain and second by the degradation of the sterol A-D rings. A compound that could disrupt the two major steps in the degradation of cholesterol in Mtb could be a great finding.

It is important to mention that spontaneous resistance to the ANB part of the T5 compound, which is similar to the PAS compound, could emerge via multiple mechanisms that include limited bio-activation within the folate synthesis pathway, efflux pumps, specific mutations like in the *pabB* gene, and an insufficient accumulation within the bacilli (Minato et al., 2015a). We do not have information about spontaneous drug-resistant mutation for the epidioxy-sterol-T1 part of the molecule. However, it is interesting that a compound like the T5 compound that acts over different metabolic pathways is less likely to encounter resistance in the same bacteria, which could be advantageous for this compound in the fight against Mtb.

The up-regulation of the ammonium transporter could be a consequence of the nitro group present in the ANB part of the T5 compound. This ANB could behave similarly to a nitroimidazole compound releasing nitric oxide (NO) and converted into ammonium by the bacteria, which has to be shuttled out to avoid toxicity. The activity that we observed for the T5 compound *in vivo* is significant but not as effective if we compared the results with drugs like Rifampicin; however, we know that the T5 compound is highly hydrophobic and may require a better vehicle in order to obtain more effective results in future animal experiments.

## Conclusion

We found a new compound called T5 with a good anti-mycobacterial activity *in vivo* and *in vitro* which may be functioning as a simultaneous inhibitor of the folate and cholesterol pathways.

## Data Availability Statement

The RNA-seq transcriptomic data was deposited in https://www.ncbi.nlm.nih.gov/bioproject: DMSO 4 h, BaenaRNA1_1.fastq.gz (SRR11195990); DMSO 20 h, BaenaRNA3_1.fastq.gz (SRR11195989); T5 4 h, BaenaRNA2_1.fastq.gz (SRR11195988); and T5 20 h, BaenaRNA4_1.fastq.gz (SRR11195987).

## Ethics Statement

The animal study was reviewed and approved by the animal ethics committee of the Universidad de Antioquia.

## Author Contributions

AB, JA, AM, and LB conceived and designed the experiments. AB, EV, and MP performed the experiments. AB, JA, MP, and EV analyzed the data. AB, LB, MP, JA, and AM wrote and revised the manuscript. All the authors read and approved the manuscript.

## Conflict of Interest

The authors declare that the research was conducted in the absence of any commercial or financial relationships that could be construed as a potential conflict of interest.

## Footnotes

1 https://biocyc.org/organism-summary?object=MTBH37RV

## Supplementary Material

FIGURE S1 Compound library and T5 NMR. **(A)** Reaction to produce the T5 compound. **(B)** ^1^H NMR spectrum of compound T5. **(C)** ^13^C NMR spectrum of compound T5. **(D)** ^1^H- ^13^C HMBC 2D NMR correlation spectroscopy of compound T5. **(E)** ^1^H- ^13^C HMBC 2D NMR correlation spectroscopy of compound T5. **(F)** Inhibitory GFP curves for the compounds T2–T4 and T6–T15 after 48 h of treatment post-infection.

FIGURE S2 CFUs of the Mtb after the THP-1 macrophage lysis. The THP-1 macrophages were lysed and 5 μl of bacteria were plated for each dilution in 7H10 media for 23 days. The plates were divided in 4 quadrants and labeled clockwise with the dilution (Undiluted [1], 10^–1^, 10^–2^, 10^–3^), starting with upper left quadrant.

FIGURE S3 RNA quality of the samples used for the RNA-seq. **(A)** Agarose gel showing the integrity of the ribosomal RNA bands. **(B)** RNA integrity number (RIN) obtained for each sample. **(C)** Showing the bio-analyzer electropherograms for each sample.

FIGURE S4 Cell viability assay quantification. **(A)** The cell death phenotypes encountered after the treatment with the different compounds. **(B)** Example of the cell death quantification procedure that we have used to generate the viability data in Figure 4B.

TABLE S1 THP-1 Macrophage transcriptome.

TABLE S2 Mtb-H37Rv transcriptome.
