# Supplementary figures and images for: New Conjugated Compound T5 Epidioxy-Sterol-ANB Inhibits the Growth of Mycobacterium tuberculosis Affecting the Cholesterol and Folate Pathways
Source: Front Microbiol. 2020 Sep 10;11:537935. doi: 10.3389/fmicb.2020.537935 (PMC7533559; doi:10.3389/fmicb.2020.537935)

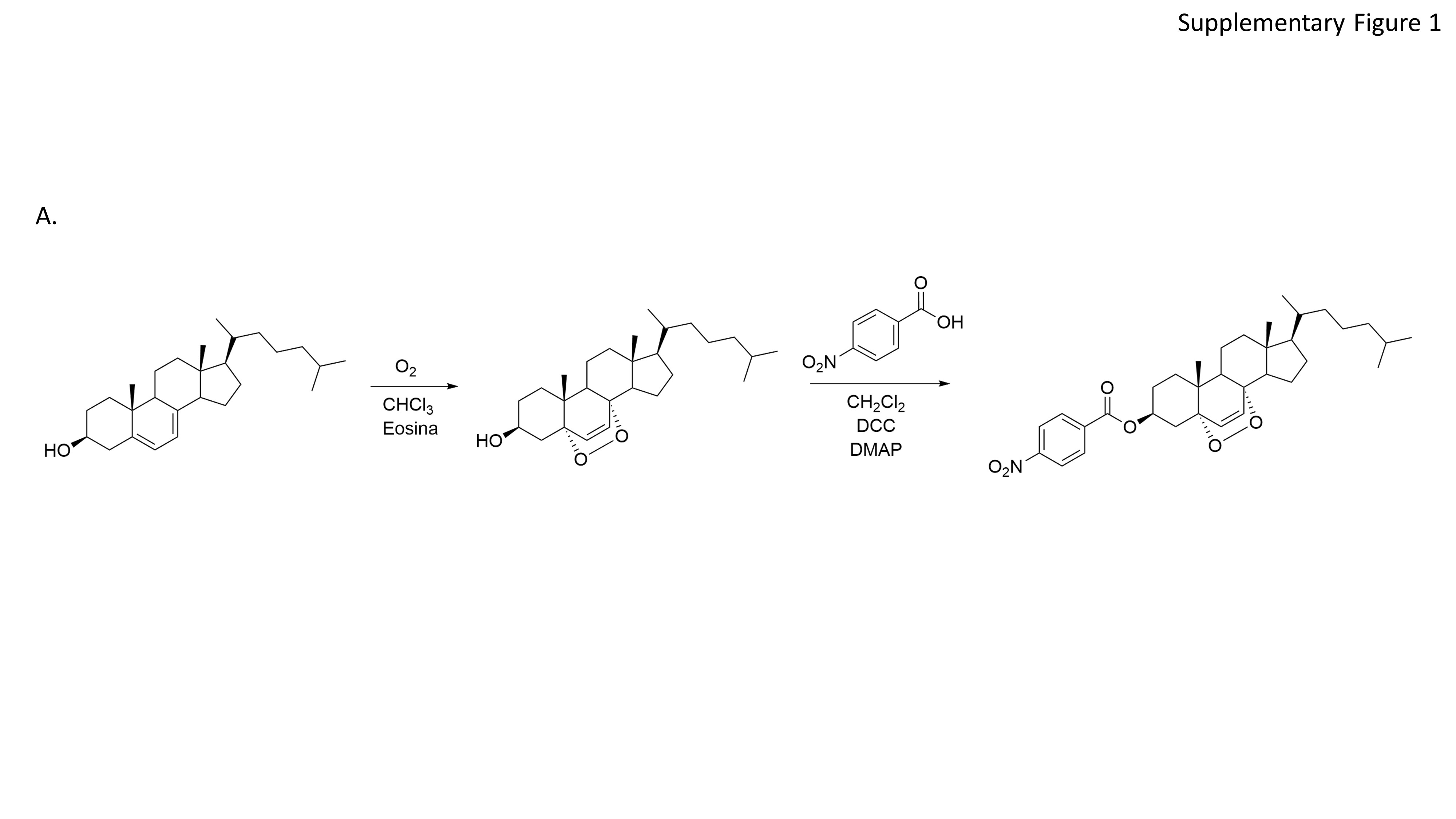

Supplement: FIGURE S1 — Compound library and T5 NMR. (A) Reaction to produce the T5 compound. (B) 1H NMR spectrum of compound T5. (C) 13C NMR spectrum of compound T5. (D) 1H- 13C HMBC 2D NMR correlation spectroscopy of compound T5. (E) 1H- 13C HMBC 2D NMR correlation spectroscopy of compound T5. (F) Inhibitory GFP curves for the compounds T2–T4 and T6–T15 after 48 h of treatment post-infection. [file Image_1.JPEG]

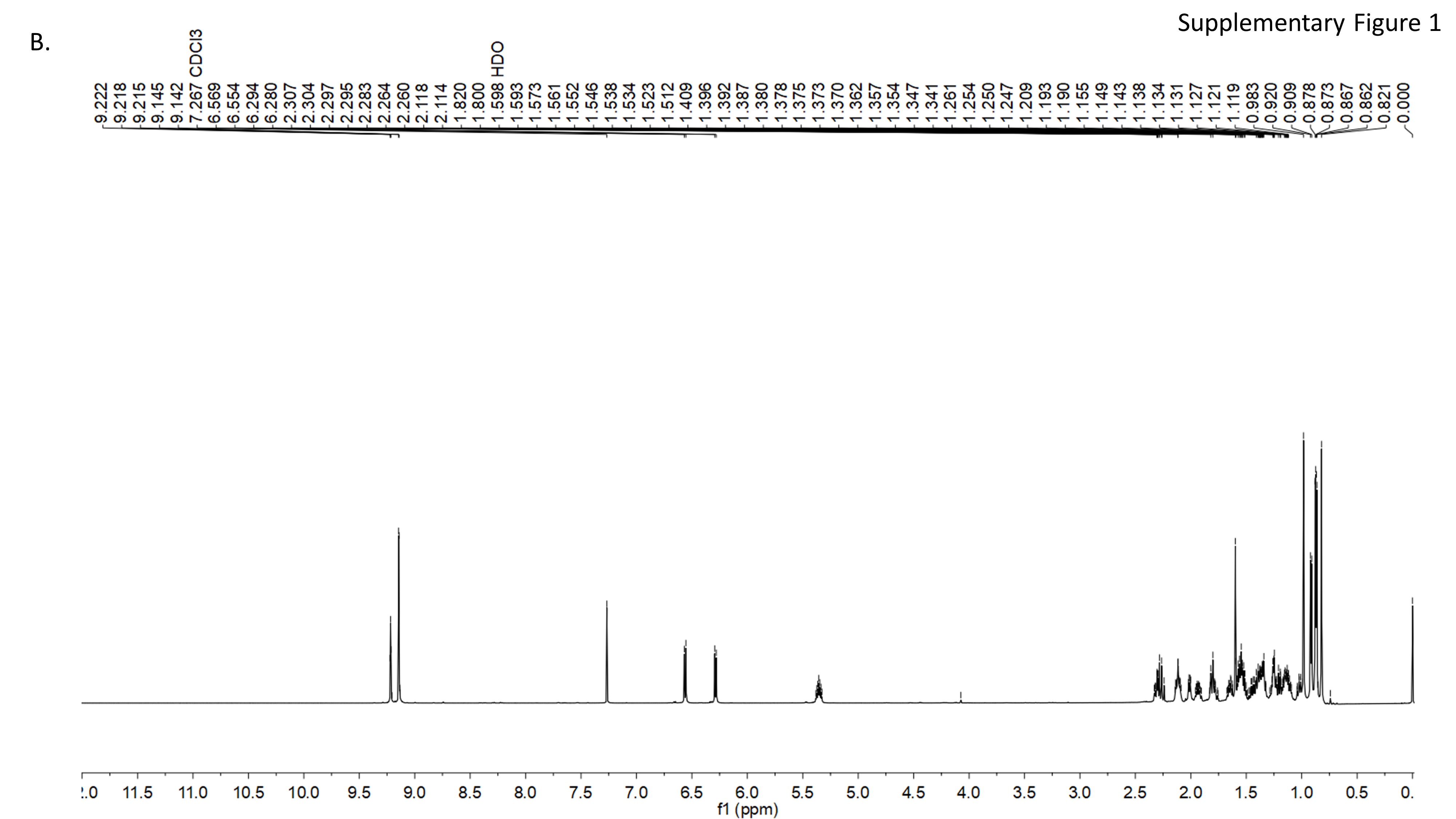

Supplement: FIGURE S2 — CFUs of the Mtb after the THP-1 macrophage lysis. The THP-1 macrophages were lysed and 5 μl of bacteria were plated for each dilution in 7H10 media for 23 days. The plates were divided in 4 quadrants and labeled clockwise with the dilution (Undiluted [1], 10–1, 10–2, 10–3), starting with upper left quadrant. [file Image_2.JPEG]

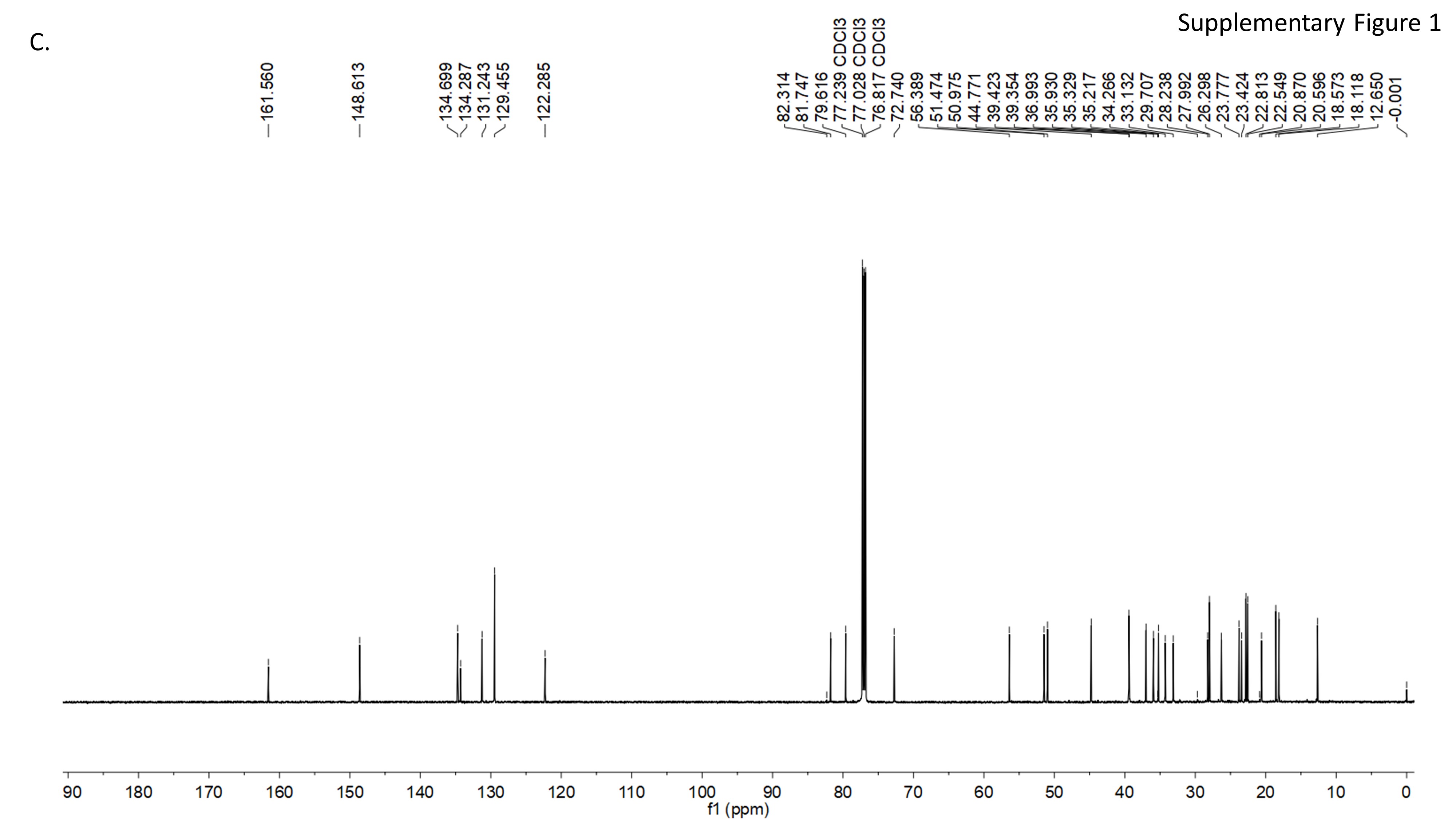

Supplement: FIGURE S3 — RNA quality of the samples used for the RNA-seq. (A) Agarose gel showing the integrity of the ribosomal RNA bands. (B) RNA integrity number (RIN) obtained for each sample. (C) Showing the bio-analyzer electropherograms for each sample. [file Image_3.JPEG]

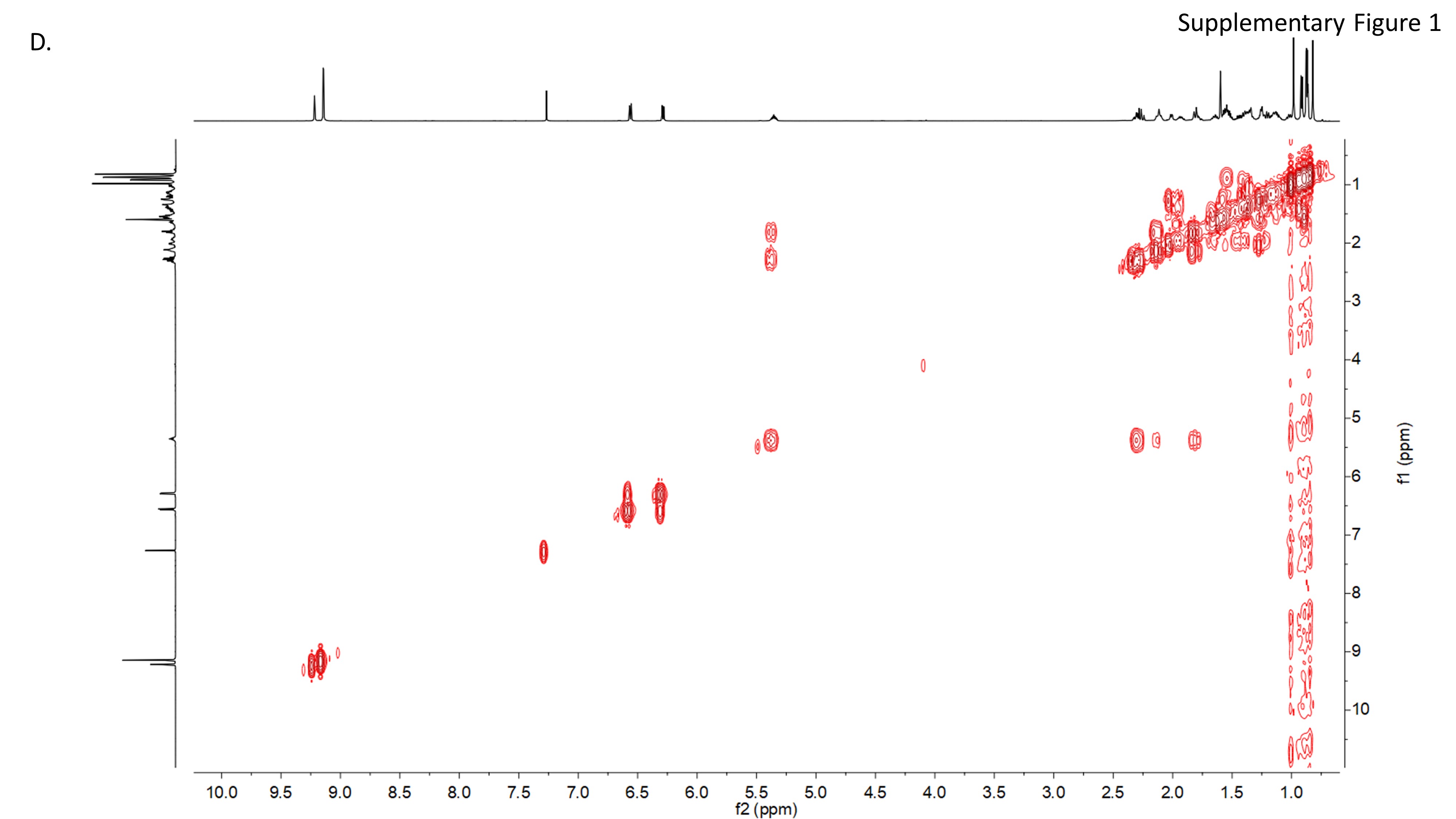

Supplement: FIGURE S4 — Cell viability assay quantification. (A) The cell death phenotypes encountered after the treatment with the different compounds. (B) Example of the cell death quantification procedure that we have used to generate the viability data in Figure 4B. [file Image_4.JPEG]

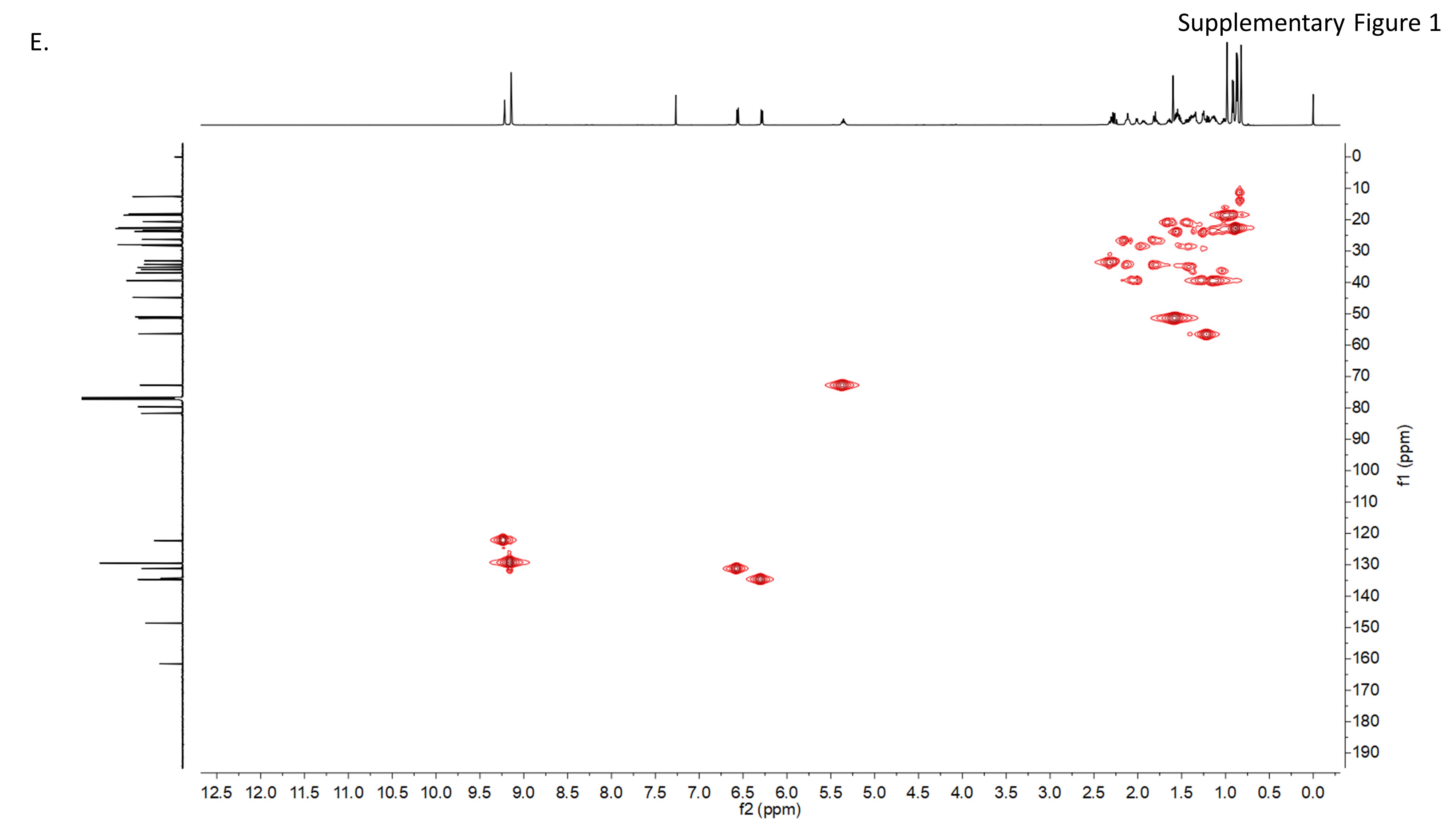

Supplement: Supplementary file 7 [file Image_5.JPEG]

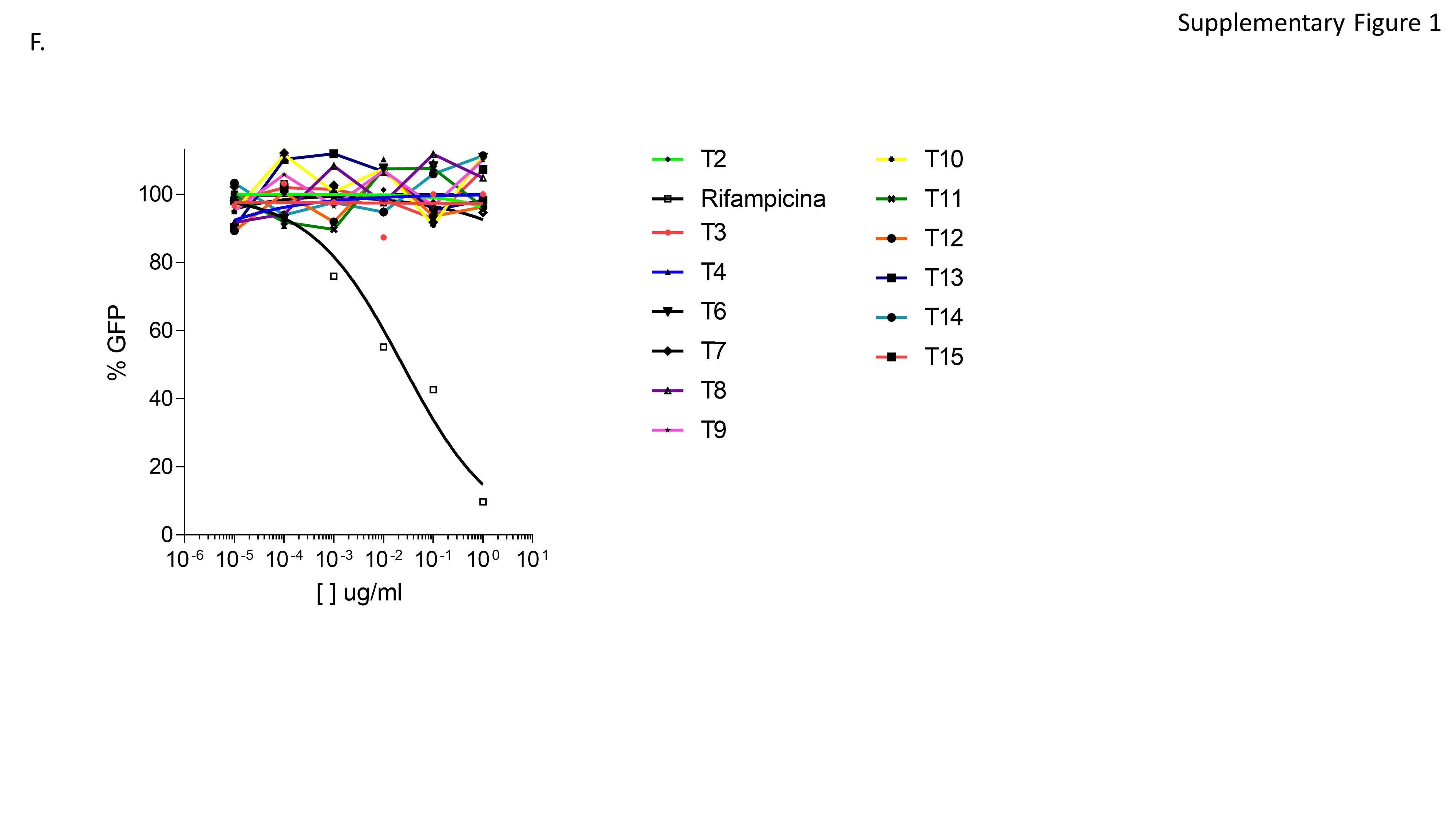

Supplement: Supplementary file 8 [file Image_6.JPEG]

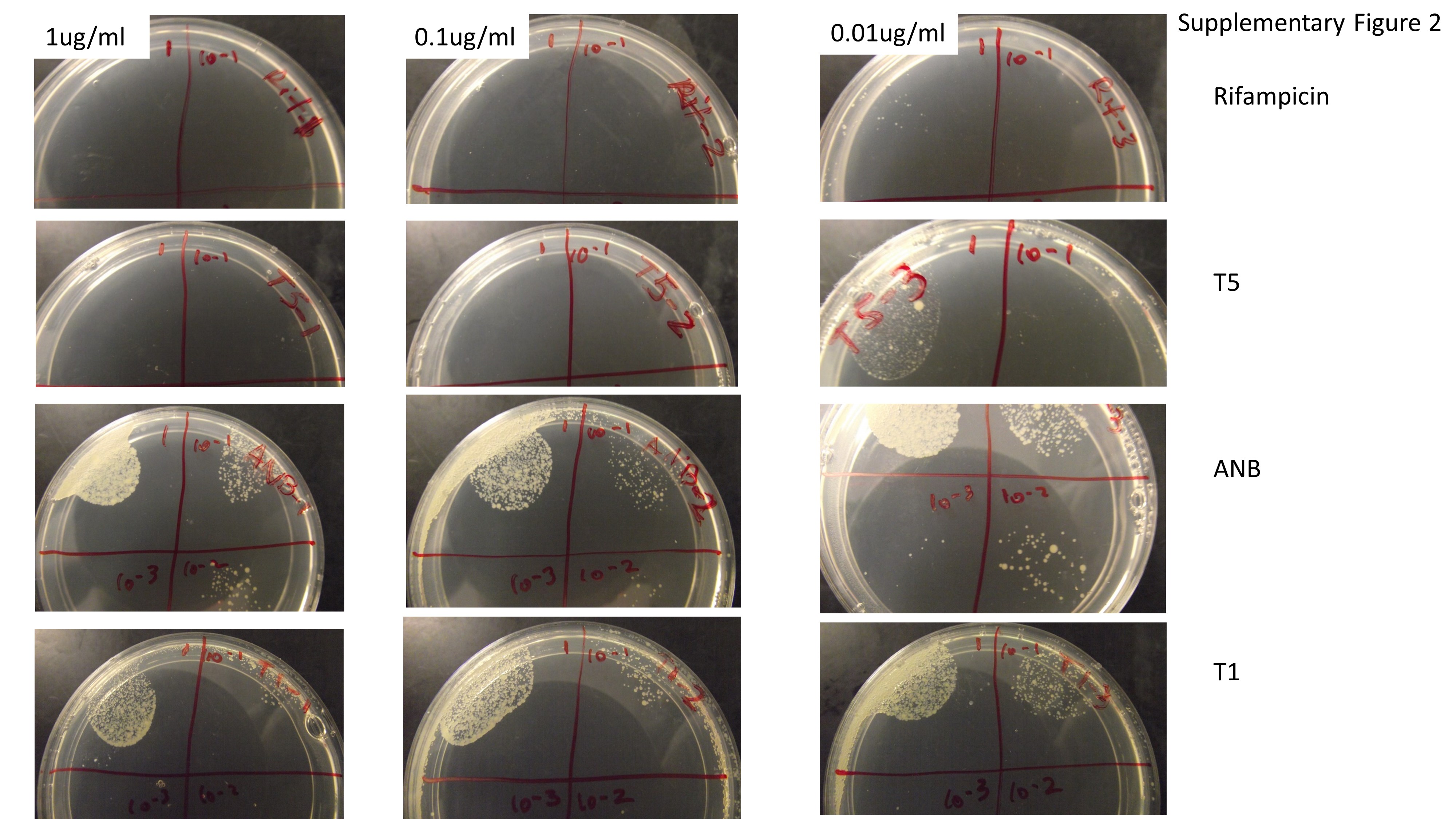

Supplement: Supplementary file 9 [file Image_7.JPEG]

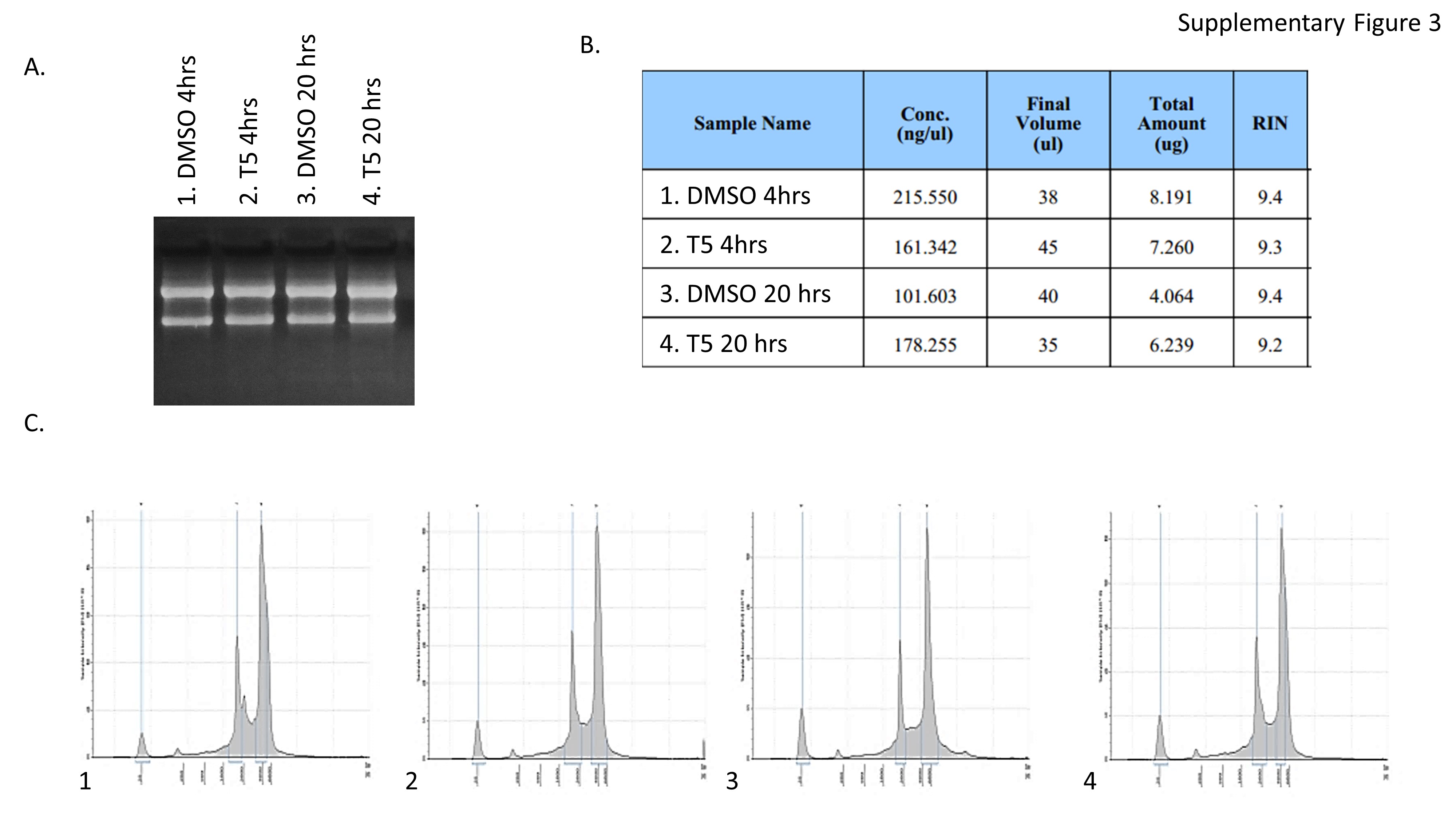

Supplement: Supplementary file 10 [file Image_8.JPEG]

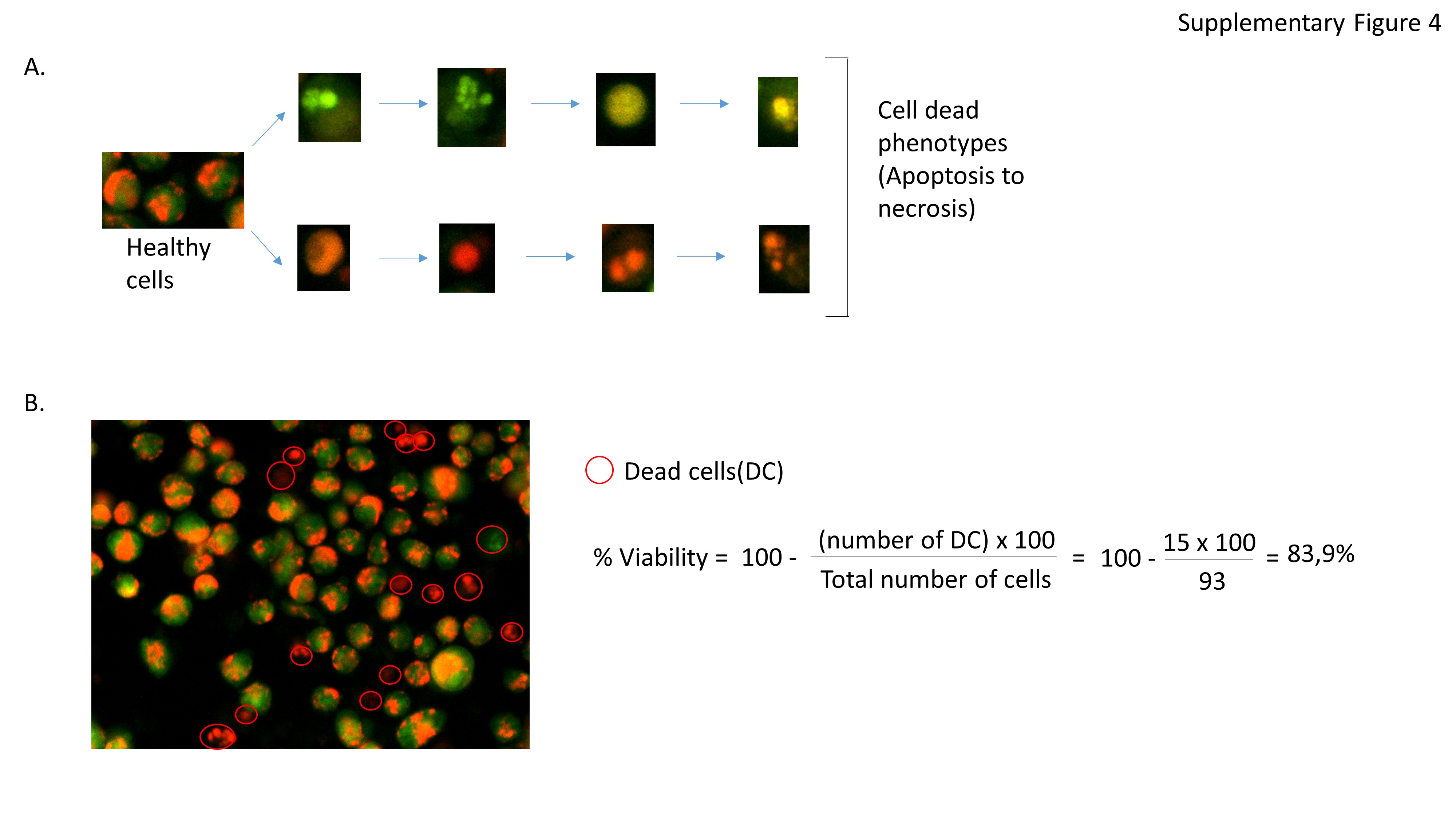

Supplement: Supplementary file 11 [file Image_9.JPEG]
